# The Effectiveness and Tolerability of Preformed Growth Factors Vehiculated Through Iontophoresis on Patients with Androgenetic Alopecia and Telogen Effluvium: A Clinical Study

**DOI:** 10.5826/dpc.1103a82

**Published:** 2021-05-20

**Authors:** Aurora Maria Alessandrini, Francesca Bruni, Bianca Maria Piraccini, Michela Starace

**Affiliations:** 1 Dermatology, Department of Experimental, Diagnostic and Specialty Medicine, University of Bologna, Italy

**Keywords:** iontophoresis, androgenetic alopecia, telogen effluvium, growth factors, scalp disorders, physical therapy

## Abstract

**Background:**

Androgenetic alopecia is characterized by a progressive miniaturization of hair follicles in a pattern distribution in genetically predisposed individuals. The efficacy of conventional therapies is variable, therefore there is a need for adjuvant and newer treatment modalities to provide faster and better outcomes.

**Objectives:**

Evaluation of vehiculated through iontopthheo ereffsiicsa icny paantdie tnotlse rwaibtihli tayn odfr ao gceonmebtiicn aeldo tpheecriaap ayn: dp raesfsoorcmiaetded g rteolwogthe nfa ecftfolursvium, to obtain faster hair regrowth.

**Materials and Methods:**

Treatment was performed between June 2018 and June 2019 on 60 patients with androgenetic alopecia and associated telogen effluvium. Each patient underwent 4 sessions in total, each session was performed every 3 weeks. Global photography and trichoscopy were collected at every session of therapy. All patients filed out a self-assessment questionnaire.

**Results:**

Results were very promising, with improvement of hair density and thickening of the hair shaft diameter in most of patients seen with both global photography and trichoscopy. All patients were satisfied of the clinical result and reported a complete reduction in hair loss. No serious adverse side effects were reported.

**Conclusions:**

The use of growth factors associated with iontophoresis technique is a useful treatment for treating and preventing androgenetic alopecia. In addition, in case of associated telogen effluvium, this technique allows for an early stop of hair shedding, especially when cosmetic procedures do not provide satisfactory results in patients.

## Introduction

Hair loss represents a problem for the patient for cosmetic and psychological reasons because hair symbolizes an important mirror of our image and a physical attractiveness to self-perception of beauty. The field of hair disorders is constantly growing. The first step to address this issue, is to collect a good historical record and perform a thorough physical examination. Laboratory testing is often unnecessary, while trichoscopy is fundamental for all hair diseases. Androgenetic alopecia and telogen effluvium are common causes of non-scarring alopecia. Many treatments are available, and a prompt diagnosis is particularly important for the prognosis.

Androgenetic alopecia (AGA) is the most common cause of non-cicatricial alopecia and affects up to 50% of women and 80% of men during their lifetime [1], with a frequency that increases with age after puberty. The disease is characterized by a progressive miniaturization of hair follicles in selected areas of the scalp, in genetically predisposed individuals. The effectiveness of conventional therapies with finasteride and minoxidil, in terms of the arrest of alopecia progression and induction of new hair regrowth, is variable between 40% and 60% [2], therefore over the years physical adjuvant treatments have been introduced to obtain faster and better results, especially for those patients who have not achieved satisfying results or that require further clinical improvement. This review highlights the importance of adding adjuvant physical or surgical therapies, such as PRP (Platelet-Rich Plasma), when standard treatments do not give enough results.

In addition, androgenetic alopecia can start with an episode of telogen effluvium (TE) characterized by a diffuse hair loss occurring around 3 months following a triggering event and lasting for about 6 months. In TE, hair loss is usually less than 50% of the scalp hair [3,4]. TE occurs more frequently in adult females.

Preformed growth factors vehiculated through iontophoresis is one of the most innovative treatments. It is a patented technique that increases hair follicle growth through 3 combined mechanisms: application of preformed growth factors with multiple microdermal incisions of the scalp, pressure wave, and iontophoresis. The use of this technique has been investigated as a potential therapeutic option for the treatment of hair disorders due to its capacity to enhance growth factor production, facilitate hair follicle development and cycling, amplify collagen and elastin production and create microchannels that allow transdermal delivery of drugs through the stratum corneum. The technique is performed through puncturing with 0,25 mm microneedles, preventing deep injury and scar formation. The procedure is sufficient to induce skin irritation and trigger skin repair mechanisms (as measured by induction of TGF-beta, TGF-alpha, FGF 7, PDGF), ultimately resulting in collagen deposition by fibroblasts. The aim of this study was to evaluate the efficacy of the application of preformed growth factors with multiple microdermal incisions of the scalp vehiculated by iontophoresis in patients with AGA and TE, supporting its combined administration along with the existing therapeutic modalities, to obtain faster hair regrowth and patient satisfaction.

## Materials and Methods

A pilot study, open-label, not randomized, single-group, and single-center was performed. 30 subjects with grade II, III, IV, and V male androgenetic alopecia according to Hamilton-Norwood Scale and 30 women of Ludwig’s grade I, II, III (15 with AGA and 15 with TE associated) second scale were included in the study between June 2018 and June 2019.

During the first enrollment visit (T0), patients underwent a dermatological examination, global photography and photomicrograph (trichoscopy and Trichoscan®). The treatment procedure included 4 sessions of microdermal incision followed by iontophoresis performed every 3 weeks. During each session, a vial containing growth factors was applied on the scalp that was subsequently treated with a skin patting device followed by iontophoresis to allow absorption of the product.

We performed two control visits, the first after 6 months from the first treatment and the second after one year. Patients were evaluated with instrumental methods for clinical and trichoscopic evaluation and self-assessment questionnaires during each visit.

No anesthesia was necessary for the procedure. The first step of this device is a controlled microdermabrasion by a sequence of micro wounds with a needle length 0,25 mm over affected areas in longitudinal, vertical, and diagonal directions, eight times in each direction or until mild erythema, which was considered as the end point to stimulate the dermis repair process resulting in increased vascularization, release of growth factors, fibroblast multiplication and increased collagen and elastin synthesis.

The device also produces a radial pressure wave (mechanical action) directed to the scalp. It has 3 different effects: strengthening of the microcirculation, stimulation of cellular metabolism that increases the intake of active ingredients, stimulation of fibroblast activity with collagen and elastin production. Finally, the iontophoresis induces a muscular stress enhancing the contractile capacity of the skin and inducing the dilation of the pores of the skin to facilitate the absorption of the active ingredients. At the end of the treatment, the scalp is irradiated with red LED light with a bio-stimulant effect on the production of fibroblasts and elastin. Each procedure lasted for about 20–25 minutes.

After the procedure, it is unnecessary to clean the scalp or apply any cream. Following the procedure, no precautions were recommended to patients, who can continue with cosmetic or pharmacological treatments as usual. We evaluated the efficacy and tolerability of this technique before starting the treatment and after 6 and 12 months through pull test, clinical iconography, and trichoscopy; digital images were obtained at 20×, 40×, and 70× magnifications at the vertex and central hairline of the scalp and both the number and the diameter of the hairs were measured with Trichoscan® software. We used a standardized grid located on the scalp at every session to correctly locate the same frontal and vertex scalp area during the treatment, using the Kang’s point or “V” point as primary reference. The “V” point is calculated by the intersection of the mid-sagittal line, and the coronal line connecting both tips of the tragus of the patient. Furthermore, we questioned all patients about local adverse effects or increase in hair loss, as well as their perception of hair growth.

## Results

Data was collected from a total of 60 patients, between June 2018 and June 2019. Among the female patients’ group, 15 suffered from AGA, and other 15 suffered from AGA and associated TE. All male patients (30) had AGA. All patients underwent 4 sessions of preformed growth factors vehiculated through iontophoresis at an interval of 3 weeks, over a total period of 12 months. All patients were Caucasian, and the mean age at the diagnosis was 39,9 (range 18–78 years). Global photography and trichoscopy revealed the typical aspect of AGA with the presence of diameter variability, peripilar signs and empty follicles in 75% of patients; the fifteen patients with associated TE showed short hairs in regrowth at trichoscopy and a positive pull test with telogen hair roots. All enrolled patients completed the study without adverse reactions or side effects. No pain or discomfort were reported by patients during the procedure and no erosion or breakage of hair shaft was noted on the affected areas.

Global photography and trichoscopy showed improvement in all 60 patients with a partial or complete reduction in hair loss, confirmed by a negative pull test, associated to the perception of a hair density improvement and a hair shaft diameter thickening. The results are listed in Table 1.

**Figure 1:**
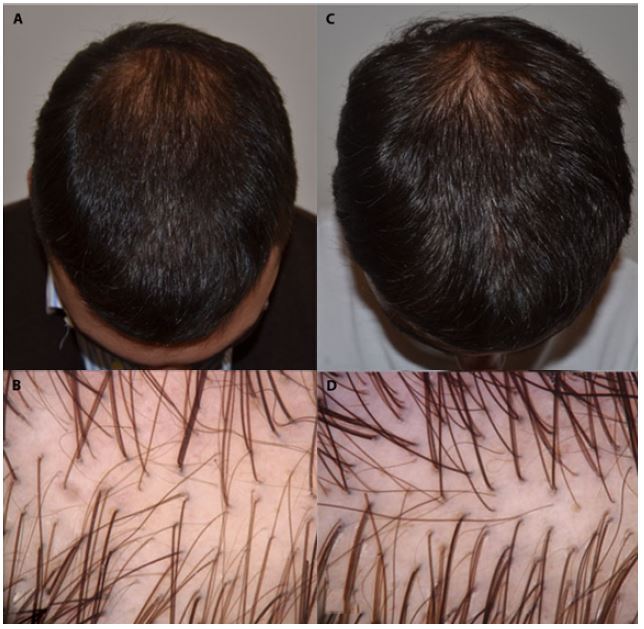
Androgenetic alopecia in a 31-y-old male. Clinical picture (A) and corresponding dermoscopic image (B) at baseline and clinical picture (C) and corresponding trichoscopic image (D) with increased hair density after 6 months.

In particular, male patients showed a 14.61% increase in the total number of hairs/cm2 in the anterior area of the scalp and a 13,62% increase in the hair diameter in the vertex area (Figure 1).

**Figure 2:**
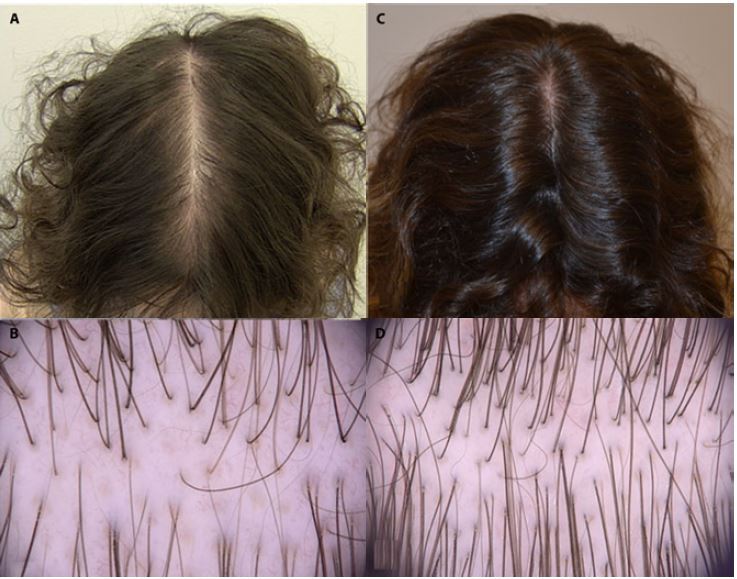
Androgenetic alopecia in a 29-y-old female. Clinical picture (A) and corresponding dermoscopic image (B) at baseline and clinical picture (C) and corresponding trichoscopic image (D) with increased hair density after 12 months.

Female patients, on the other hand, showed a 13.68% increase in the total number of hairs/cm2 at the anterior area and an increase in the hair diameter of 15,61% in the vertex area (Figure 2).

In all patients a reduction of vellus hair was detected in all areas of the scalp with an increase in the total number of hairs. After 1 year, the researcher’s evaluation reported an evident improvement in all patients: more in detail, a moderate improvement was reported in 6 patients and a significant improvement in 12. All patients were satisfied by the treatment; 17 referred a moderate improvement and 43 reported a significant improvement. Most of them (53/60) defined the treatment as “painless and pleasant”.

## Discussion and Conclusions

Androgenetic alopecia (AGA) is the most common cause of non-scarring alopecia, affecting up to 50% of women and 80% of men [1], with a frequency increasing with age after puberty. Its prevalence is higher in Caucasians than in blacks and Asians [5,6].

AGA is characterized by progressive hair thinning developing under the influence of a testosterone metabolite, dihydrotestosterone (DHT), against a background of genetically determined susceptibility of the hair follicles, in frontal, temporal and vertex regions.

Clinical manifestations are different in both sexes. In males, AGA determines a progressive fronto-temporal recession and a vertex loss, while in women the frontal hairline is preserved and hair loss involves more or less uniformly the frontal region, posteriorly to the hairline. Female patterns might occur in males and vice versa. Male AGA is commonly evaluated using the Hamilton-Norwood scale that distinguishes 12 degrees of severity. Female AGA is evaluated either using the Ludwig scale (3 stages), or the Sinclair (5 stages) or Savin scales (6 stages).

Pull test typically shows telogen roots, but trichoscopy is the most important tool for diagnosis. Androgenetic alopecia is a slowly progressing disease that, if not treated, induces diffuse hair thinning in androgen-sensitive areas of the scalp.

According to the most recent European Guidelines [2], effective medical treatments, such as finasteride and minoxidil, are available with evidence level 1, but as is well known, they are chronic therapies that may lose effectiveness over time. Many topical and systemic treatments are available. Minoxidil still represents a milestone as a “hair growth stimulator”, even if the precise mechanism of its action is not completely understood [7]. To maintain efficacy treatment should be continuous and not suspended. Over the years several types of physical treatments have assumed an important adjuvant role, especially for those patients who have not obtained satisfactory results with medical therapies or that desire further improvements. Other treatment options include the platelet-rich plasma (PRP) treatment [8,9], low-level laser (light) therapy [10], and surgery [11,12].

In addition, one of the first symptoms reported by patient is an initial hair shedding or telogen effluvium. The term telogen effluvium (TE) defines a diffuse hair loss that occurs around 3 months following a triggering event, lasting for about 6 months. TE results from noxious events that precipitate the entry of a large number of follicles into the telogen phase. Possible causes include systemic diseases, drugs, fever, stress, weight loss, delivery, iron deficiency, and inflammatory scalp disorders. Hair loss is usually less than 50% of the scalp hair [3,4]. TE is more frequent in adult females and can be the consequence of an interruption of the follicular cycle with a sudden shift from the growth (anagen) phase to the rest (telogen) phase [13]. In fact, an episode of telogen effluvium can show a consequent androgenetic alopecia in predisposed subjects.

We performed a study to evaluate the efficacy and tolerability of preformed growth factors applied through with iontophoresis for the treatment of androgenetic alopecia in 60 patients, 30 male and 30 females, for a period of 12 months. The evaluation was both subjective, based on an efficacy and tolerability questionnaire filled out by both the clinician and the patient, and objective, through the comparison of global photographs and serial photomicrographs (trichoscopy at 20X, 40X, 70X and Trichoscan® magnifications (FotoFinderdermoscope, Teachscreen Software, Bad Birnbach, Germany). Our study illustrated the efficacy and non-invasiveness of a treatment procedure with preformed growth factors through iontophoresis.

This technique works by increasing the blood flow to hair follicles, stimulating stem cells and inducing the activation of growth factors by neovascularization and neocollagenesis. As reported in the literature, numerous growth factors can stimulate the hair growth cycle [14–17]. VEGF, essential for angiogenesis and vascular permeability, is responsible for maintaining the correct vascularization of the hair follicle in the anagen phase. IGF-I promotes growth by regulating cell proliferation and migration during the development of hair follicles. B-FGF promotes the anagen phase in hair follicles and is considered a potential promoter of hair growth. KGF is essential for regenerating hair follicles by stimulating more resistant stem growth. Finally, EGF has a direct action on fibroblasts enhancing their action on collagen and elastin production.

Treatment to arrest alopecia progression and induce new hair regrowth in androgenetic alopecia patients include finasteride and minoxidil. Oral intake of nutritional supplements containing iron, vitamins, and aminoacids, and topical application of cosmetic lotions formulated to block acute hair shedding and promote hair growth [18] include Insulin-like growth factor 1 (IGF-1), Fibroblast growth factor (FGF), and Vascular Endothelia Growth Factor (VEGF).

Adjuvant and recent treatments include physical therapies such as PRP or microneedling [19] where there is an improvement in hair growth through the stimulation of dermal papilla and stem cells and an increase in hair follicles blood supply with growth factors recruitment. However, these techniques are often reported as painful by the patients, and in some cases the pain is hard to bear.

Our study confirms the fundamental role of the association between the use of growth factors conveyed associated with the iontophoresis technique in increasing hair regrowth and hair diameter avoiding pain or discomfort symptoms. This technique represents a safe and useful option to treat androgenetic alopecia, especially when associated with telogen effluvium, through mechanisms that include stimulation and elongation of hair follicle anagen phase, increased blood microcirculation, activation of fibroblasts with collagen, and elastin production. Furthermore, this procedure is simple to perform and extremely pleasant for the patient. Future large controlled clinical trials exploring the utility of preformed growth factors through iontophoresis are imperative to prove its validation as an evidence-based therapeutic option for patients with a variety of hair disorders, thus confirming its role as more than a cosmeceutical treatment.

## Figures and Tables

**Table 1:** Results of our study.

	T0	T6	Difference% (T0–T6)
*Female patients*			
Anterior average hair density	163,96	186,39	13,68%
Vertex average hair density	161,22	188,95	17,20%
Anterior average vellus hair/cm2	41,43	33,71	−0,62%
Vertex average vellus hair/cm2	34,21	28,95	−0,51%
Anterior average hair diameter	0,08	0,09	14,28%
Vertex average hair diameter	0,08	0,10	15,61%
Average Pull test	6,37	2,60	−59,16%
*Male patients*			
Anterior average hair density	151,00	173,06	14,61%
Vertex average hair density	146,44	169,44	15,70%
Anterior average vellus hair/cm2	38,77	35,97	−7,23%
Vertex average vellus hair/cm2	38,44	34,85	−9,32%
Anterior average hair diameter	0,07	0,08	8,67%
Vertex average hair diameter	0,07	0,08	13,62%
Average Pull test	4,60	2,47	−46,38%
